# Clinical characteristics and in-hospital outcomes of pneumothorax in pneumoconiosis: a tertiary referral cohort study

**DOI:** 10.3389/fmed.2026.1834578

**Published:** 2026-04-29

**Authors:** Ping-Yang Hong, Jing-Huang Cai, Yu-Bing Yan, Jing-Rui Lu, Nai-Shan Zheng, Ling Cai, Yi-Li Lin, Mao-Hong Huang, Xiao-Bin Zhang

**Affiliations:** 1 Department of Pulmonary and Critical Care Medicine, School of Medicine, Zhongshan Hospital of Xiamen University, Xiamen University, Xiamen, Fujian, China; 2 The School of Clinical Medicine, Fujian Medical University, Fuzhou, Fujian, China; 3 Key Laboratory of Sleep Medicine, The Second Affiliate Hospital of Fujian Medical University, Fujian Province Universities, Quanzhou, Fujian, China; 4 Department of Medical Insurance and Price Management, School of Medicine, Zhongshan Hospital of Xiamen University, Xiamen University, Xiamen, Fujian, China; 5 Department of General Medicine, School of Medicine, Zhongshan Hospital of Xiamen University, Xiamen University, Xiamen, Fujian, China

**Keywords:** bronchoscopy, chest tube drainage, pneumoconiosis, pneumothorax, prognosis

## Abstract

**Background:**

Pneumoconiosis is a progressive occupational lung disease often complicated by secondary spontaneous pneumothorax, but detailed clinical data from tertiary centers are limited.

**Methods:**

We retrospectively analyzed 42 patients with pneumoconiosis and pneumothorax admitted to a tertiary university-affiliated hospital in China from January 2020 to July 2025. Clinical characteristics, laboratory and imaging findings, management strategies, and in-hospital outcomes were analyzed, with predefined subgroup comparisons between first-time and recurrent pneumothorax.

**Results:**

The cohort included 42 patients (40 men and 2 women) with a mean age of 57.4 years; most were smokers (73.8%), and nearly all had stage III pneumoconiosis (92.9%). Right-sided pneumothorax was more common than left-sided or bilateral disease, and pulmonary infection was noted in 76.2% of patients. Chest tube drainage was the primary treatment (88.1%), while pleurodesis, medical thoracoscopy, and bronchoscopic occlusion were used in selected cases. Patients with recurrent pneumothorax had significantly longer chest tube duration (median 15.5 vs. 4.5 days; U = 47.0, *p* = 0.0043) and hospital stays (19.5 vs. 9.0 days; U = 41.0, *p* = 0.0024) compared with first-time cases. Overall, 88.1% of patients improved and were discharged, with no in-hospital deaths.

**Conclusion:**

Pneumothorax in pneumoconiosis, typically occurring in advanced disease and often complicated by infection, is frequently associated with prolonged chest tube drainage, highlighting the importance of early recognition, individualized management, and infection control.

## Introduction

Pneumoconiosis is a chronic interstitial lung disease caused by prolonged inhalation of mineral dust, commonly seen in individuals with long-term occupational exposure to coal, silica, or other inorganic particles (1, 2). Despite advances in workplace safety, pneumoconiosis remains a significant health burden in many industrial regions (3, 4). One of the most serious complications in patients with advanced pneumoconiosis is secondary spontaneous pneumothorax (5, 6), which can acutely decompensate respiratory function and substantially increase morbidity and mortality (6).

Structural lung destruction, including extensive fibrosis, bullae formation, and pleural thickening, predisposes these patients to alveolar rupture and pleural air leakage (7). In addition, the compromised pulmonary reserve (8) in advanced-stage disease makes even a modest pneumothorax clinically significant. Prompt diagnosis and tailored management are essential to prevent respiratory failure and improve patient outcomes (9).

Previous studies have documented pneumothorax in various respiratory conditions (6, 10); however, literature focusing on pneumoconiosis-related pneumothorax, especially in complex cases or case with persistent air leaks (PAL >7 days), remains limited. Moreover, little is known about the real-world use of advanced bronchoscopic or medical thoracoscopy in these patients. In particular, detailed clinical data from tertiary referral centers remain scarce, especially in patients with advanced-stage pneumoconiosis who often require complex interventional management such as medical thoracoscopy or bronchoscopic occlusion.

The present study retrospectively reviewed 42 patients with pneumoconiosis complicated by pneumothorax treated at a tertiary university-affiliated hospital in China. The objectives were to characterize their clinical features and management patterns, and to explore factors associated with prolonged drainage and hospitalization by comparing first-time and recurrent pneumothorax. By focusing on a high-acuity referral population, this study provides clinically relevant real-world evidence to support risk stratification and management decisions in pneumoconiosis-related pneumothorax.

## Methods

### Study design and setting

We conducted a retrospective observational study at a tertiary university-affiliated hospital in China between January 2020 and July 2025. As a referral center for advanced pneumoconiosis, the hospital routinely manages complex cases transferred from secondary or community hospitals. The study protocol was approved by the Ethics Committee of Zhongshan Hospital of Xiamen University, Xiamen, China (approval no.2020–023). The requirement for informed consent was waived by the Ethics Committee of Zhongshan Hospital, Xiamen University, due to the retrospective nature of the study. All methods were performed in accordance with the relevant guidelines and regulations, including the Declaration of Helsinki and local institutional policies. All patient information was anonymized prior to analysis to protect privacy and confidentiality.

### Patient selection

**Figure 1:**
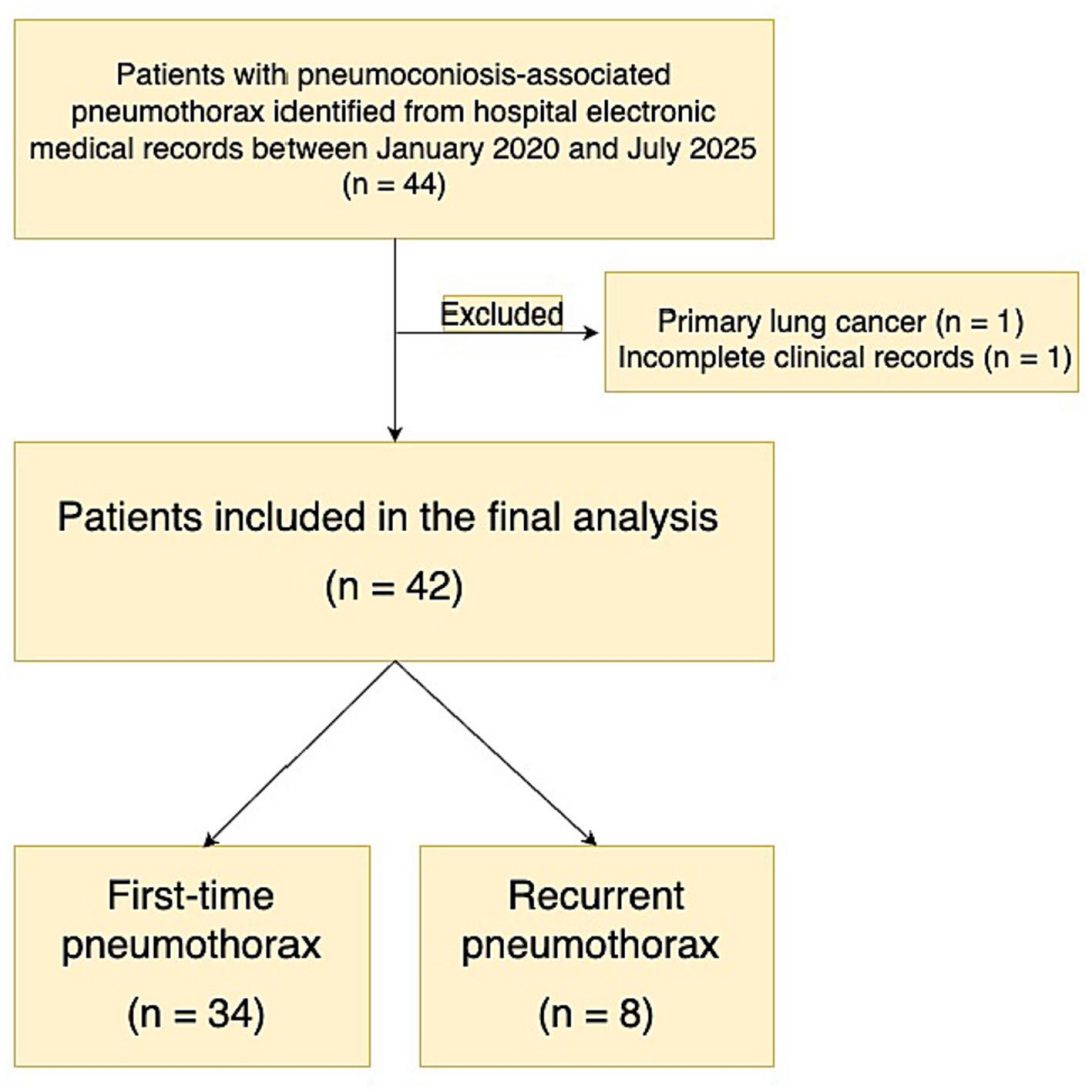
Flow diagram of patient selection. Patients with pneumoconiosis-associated pneumothorax were identified from hospital electronic medical records between January 2020 and July 2025. After exclusion of patients with primary lung cancer (*n* = 1) and incomplete clinical records (*n* = 1), a total of 42 patients were included in the final analysis and categorized into first-time (*n* = 34) and recurrent pneumothorax groups (*n* = 8).

Eligible patients were identified through electronic medical records (Figure 1). Inclusion criteria were: (1) age ≥18 years, (2) confirmed diagnosis of pneumoconiosis based on occupational history and radiologic findings, and (3) radiologically confirmed pneumothorax by chest radiograph or CT. Patients with primary lung cancer, severe cardiac dysfunction, or incomplete records were excluded. The diagnosis and staging of pneumoconiosis were based on the Chinese national diagnostic criteria (GBZ 70–2015) (11).

### Data collection

Clinical and demographic data were extracted using standardized forms and included age, sex, smoking history, body mass index, occupational exposure history, pneumoconiosis subtype and stage, and history of tuberculosis. Pneumothorax characteristics were recorded with respect to laterality, type, whether it was a first episode or recurrence, and estimated size. The size of pneumothorax was estimated based on radiologic evidence of lung collapse documented in the medical records and categorized broadly according to British Thoracic Society criteria, in which a large pneumothorax is defined as a visible rim of ≥2 cm between the lung margin and chest wall at the level of the hilum on chest radiograph (12). Laboratory values comprised arterial blood gas results (pH, PaO₂ and PaCO₂) and complete blood counts. These laboratory and arterial blood gas measurements primarily represented values obtained at admission or during the early stage of hospitalization when pneumothorax was first evaluated. However, FiO₂ at the time of sampling was not consistently documented in the medical records. Computed tomography scans were reviewed for evidence of fibrosis, bullae, pleural thickening, emphysematous changes, and infection-related changes such as consolidation or cavitation. Pulmonary infection was defined according to established Chinese clinical practice guidelines (13), based on a combination of clinical manifestations, laboratory findings, and radiologic evidence documented in the medical records. Microbiological confirmation was reviewed when available but was not consistently obtained in all patients due to the retrospective study design. Therefore, the diagnosis of pulmonary infection was primarily based on clinical and radiologic criteria documented in the medical records. Moreover, the timing of infection onset relative to interventional procedures could not be consistently determined due to the retrospective study design. Treatment strategies included oxygen therapy, chest tube drainage, pleurodesis, bronchoscopic occlusion techniques, and medical thoracoscopy, with details of intervention type, complications, and need for escalation of care documented. Management strategies were generally guided by established clinical practice recommendations (14) for secondary spontaneous pneumothorax, including the British Thoracic Society Pleural Disease Guideline (12), and were individualized according to persistent air leaks, recurrence, lung re-expansion status, and overall clinical condition. Specifically, pleurodesis was considered in patients with persistent air leaks or high risk of recurrence who were not suitable surgical candidates; medical thoracoscopy was performed when chest tube drainage failed to achieve adequate lung re-expansion or when bullae or pleural adhesions required direct intervention; and bronchoscopic occlusion was mainly applied in patients with persistent air leaks who were poor candidates for surgery because of severely impaired pulmonary function. Clinical outcomes included chest tube duration, length of hospital stay, pneumothorax resolution, recurrence during hospitalization, discharge status, and in-hospital mortality. Patients were categorized into first-time pneumothorax and recurrent pneumothorax groups according to whether they had a documented history of pneumothorax prior to the index hospitalization. Data extraction was performed independently by two trained investigators, and radiologic interpretations were verified by two thoracic radiologists.

### Statistical analysis

Continuous variables were expressed as mean ± standard deviation or median with interquartile range, as appropriate, and categorical variables as counts and percentages. Between-group comparisons were performed using Student’s t-test or Mann–Whitney U test for continuous variables, and chi-square test or Fisher’s exact test for categorical variables. A two-sided *p* value of <0.05 was considered statistically significant, and analyses were performed using SPSS version 22.0 (IBM Corp., Armonk, NY).

## Results

A total of 42 patients with pneumoconiosis complicated by pneumothorax were identified and included in this study. Among these, 40 (95.2%) were male and 2 (4.8%) were female, with a mean age of 57.4 ± 7.8 years (range, 43–70 years). Thirty-one patients (73.8%) had a documented history of smoking, with a median smoking burden of 30 pack-years, whereas seven patients (16.7%) denied smoking and four patients had incomplete records regarding smoking status.

Most patients had a long history of occupational dust exposure, typically more than 20 years, and presented with advanced pneumoconiosis at the time of admission. Staging revealed that 39 patients (92.9%) had stage III pneumoconiosis, 2 patients (4.8%) had stage II disease, and only 1 patient (2.4%) had stage I disease. Pneumoconiosis subtypes included mixed dust pneumoconiosis in 31 patients (73.8%) and silicosis in 11 patients (26.2%). Six patients (14.3%) also had a history of pulmonary tuberculosis, as confirmed by clinical documentation or imaging.

Most patients underwent lung imaging examinations due to dyspnea. All included patients had radiologically confirmed pneumothorax. Secondary spontaneous pneumothorax was the predominant type, reflecting the underlying structural lung damage. Right-sided pneumothorax (27/42, 64.3%) was more common than left-sided involvement (13/42, 31.0%), and bilateral cases (2/42, 4.8%) were rare but associated with more severe symptoms. The majority of patients experienced radiologically large pneumothorax according to British Thoracic Society criteria with radiological evidence of significant lung compression (Table 1).

**Table 1:** Baseline characteristics of patients with pneumoconiosis and pneumothorax (*N* = 42).

Characteristic	Numbers (*n*/%)
Gender	
Male	40 (95.2%)
Female	2 (4.8%)
Age	
40–49 years	8 (19.0%)
50–59 years	20 (47.6%)
60–69 years	11 (26.2%)
Age ≥ 70 years	3 (7.1%)
Median age, years	56.5 (52.0–64.0)
Smoking history	
Current/former	31 (73.8%)
Never	7 (16.7%)
Unknown	4 (9.5%)
Smoking burden (pack-years)	
10 ≥ Smoking burden	11 (26.2%)
11–20	7 (16.7%)
21–30	9 (21.4%)
31–40	5 (11.9%)
Smoking burden ≥ 40	6 (14.3%)
Unknown	4 (9.5%)
Pneumoconiosis stage	
Stage I	1 (2.4%)
Stage II	2 (4.8%)
Stage III	39 (92.9%)
Pulmonary tuberculosis history	
Positive	6 (14.3%)
Negative	36 (85.7%)
Laterality of pneumothorax	
Right	27 (64.3%)
Left	13 (31.0%)
Bilateral	2 (4.8%)
CT findings*	
Fibrosis^#^/Interstitial changes	12 (28.6%)
Pleural thickening	9 (21.4%)
Bullae/Emphysema	20 (47.6%)
Calcified lymph nodes	3 (7.1%)
Hospital stay, days	
5 > Hospital stay	8 (19.5%)
6–10	14 (34.1%)
11–15	11 (26.8%)
16–20	4 (9.8%)
21–29	4 (9.8%)
Hospital stay ≥ 30	1 (2.4%)
Median hospital stay, days	10 (6–15)

*CT imaging findings were not mutually exclusive; percentages were calculated based on the total number of patients, and individual patients could present with more than one radiologic feature. ^#^Fibrosis refers to explicitly reported fibrotic bands or localized fibrotic changes on CT imaging rather than the diffuse background fibrosis inherent to pneumoconiosis staging, which is reflected separately by disease stage classification.

**Figure 2:**
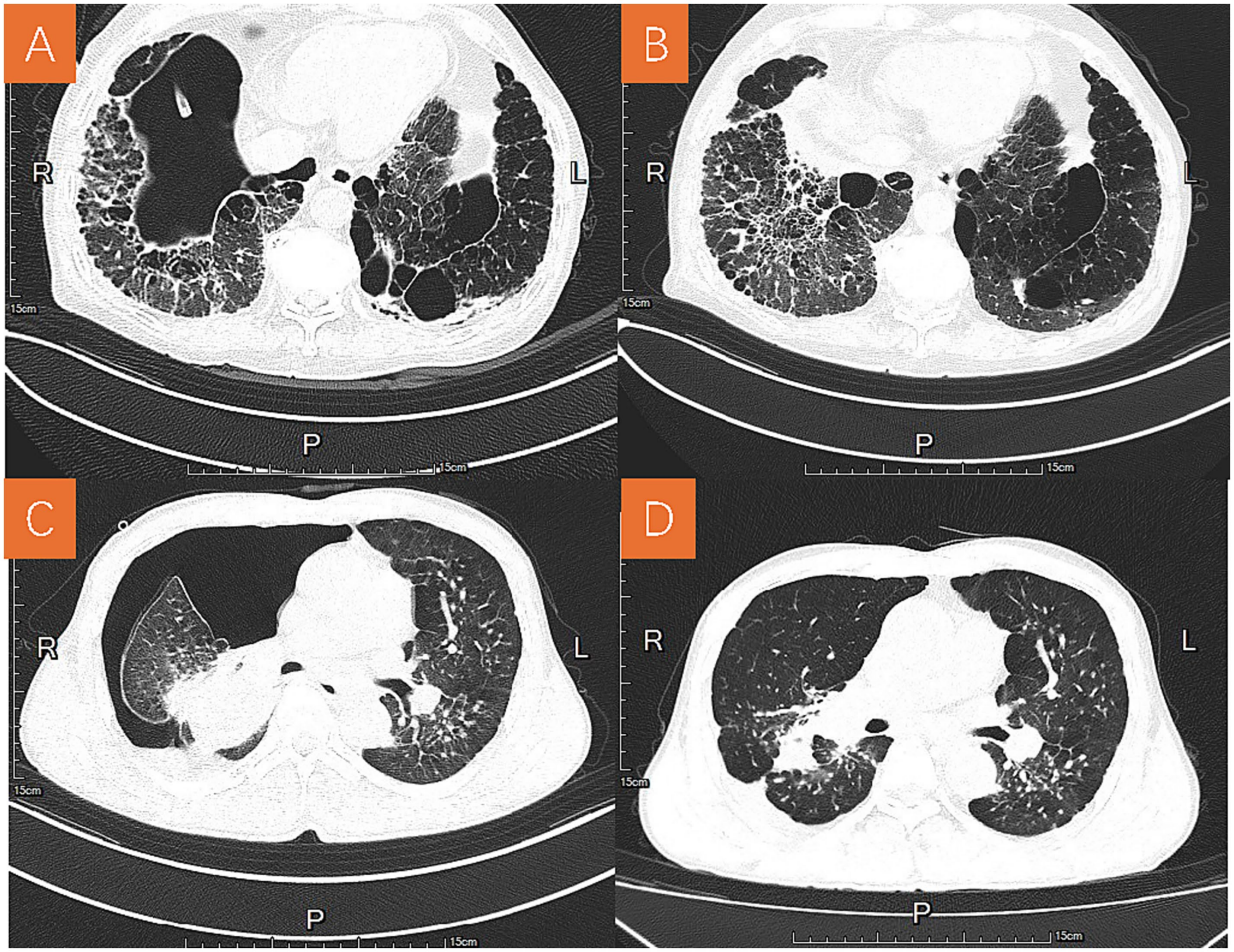
Representative chest CT images before and after treatment in two patients with pneumoconiosis-associated pneumothorax. **(A)** Chest CT images of patient A before treatment. **(B)** Chest CT images of patient A after treatment. **(C)** Chest CT images of patient B before treatment. **(D)** Chest CT images of patient B after treatment, showing improvement of pneumothorax and lung re-expansion.

CT reports consistently demonstrated diffuse fibrotic changes, pleural thickening, and calcified lymph nodes in the hilar or mediastinal regions. Compared with secondary spontaneous pneumothorax (SSP) in non-pneumoconiosis patients, pneumothorax associated with pneumoconiosis demonstrated more pronounced pleural adhesions and irregular pneumothorax cavities on CT. In several cases, encapsulated pneumothorax was observed, requiring multiple drainage catheters at different sites to achieve re-expansion of the lung (Figure 2).

Pulmonary bullae and emphysematous changes were observed in many patients, serving as likely anatomical sources of alveolar rupture. In 32 patients (76.2%), radiologists noted concurrent findings suggestive of pulmonary infection, including consolidation, tree-in-bud changes, or cavity formation. Pulmonary function tests were not performed during hospitalization in any patient due to the presence of pneumothorax.

Arterial blood gas analysis and hematologic testing revealed variable abnormalities (Table 2). The median arterial pH was 7.422 (IQR, 7.398–7.448), with acidosis (pH < 7.35) observed in 2 patients (4.8%). The median PaCO₂ was 37.8 mmHg (IQR, 35.6–42.9), and hypercapnia (PaCO₂ > 45 mmHg) occurred in 6 patients (14.3%). The median PaO₂ was 86.6 mmHg (IQR, 69.2–104.4), while 12 patients (28.6%) had hypoxemia (PaO₂ < 80 mmHg). However, because FiO₂ at the time of arterial blood gas sampling was not consistently documented, these PaO₂ values should not be interpreted as standardized measures of oxygenation. The median white blood cell count was 8.43 × 10^9^/L (IQR, 7.16–9.86), with 8 patients (19.0%) showing leukocytosis (>10 × 10^9^/L). The median hemoglobin concentration was 135 g/L (IQR, 125.5–143), and anemia (Hb < 120 g/L) was present in 6 patients (14.3%). Platelet counts were generally preserved, with a median of 245 × 10^9^/L (IQR, 197.5–277) and no cases of thrombocytopenia (<100 × 10^9^/L) (Table 2).

**Table 2:** Laboratory and arterial blood gas findings in patients with pneumoconiosis and pneumothorax (*N* = 42).

Parameter*	Finding	Numbers (*n*/%)	Median (IQR)
Arterial pH	Mild–moderate acidosis (pH < 7.35)	2 (4.8%)	7.422 (7.398–7.448)
PaCO₂	Elevated (>45 mmHg)	6 (14.3%)	37.8 (35.6–42.9) mmHg
PaO₂	Reduced (<80 mmHg)	12 (28.6%)	86.6 (69.2–104.4) mmHg
White blood cell count	Elevated (>10 × 10^9^/L)	8 (19.0%)	8.43 (7.16–9.86) × 10^9^/L
Hemoglobin	<120 g/L (anemia)	6 (14.3%)	135 (125.5–143) g/L
Platelet count	<100 × 10^9^/L (thrombocytopenia)	0 (0%)	245 (197.5–277) × 10^9^/L

*Laboratory and arterial blood gas values represent measurements obtained at admission or early during hospitalization. IQR, Interquartile Range.

**Figure 3:**
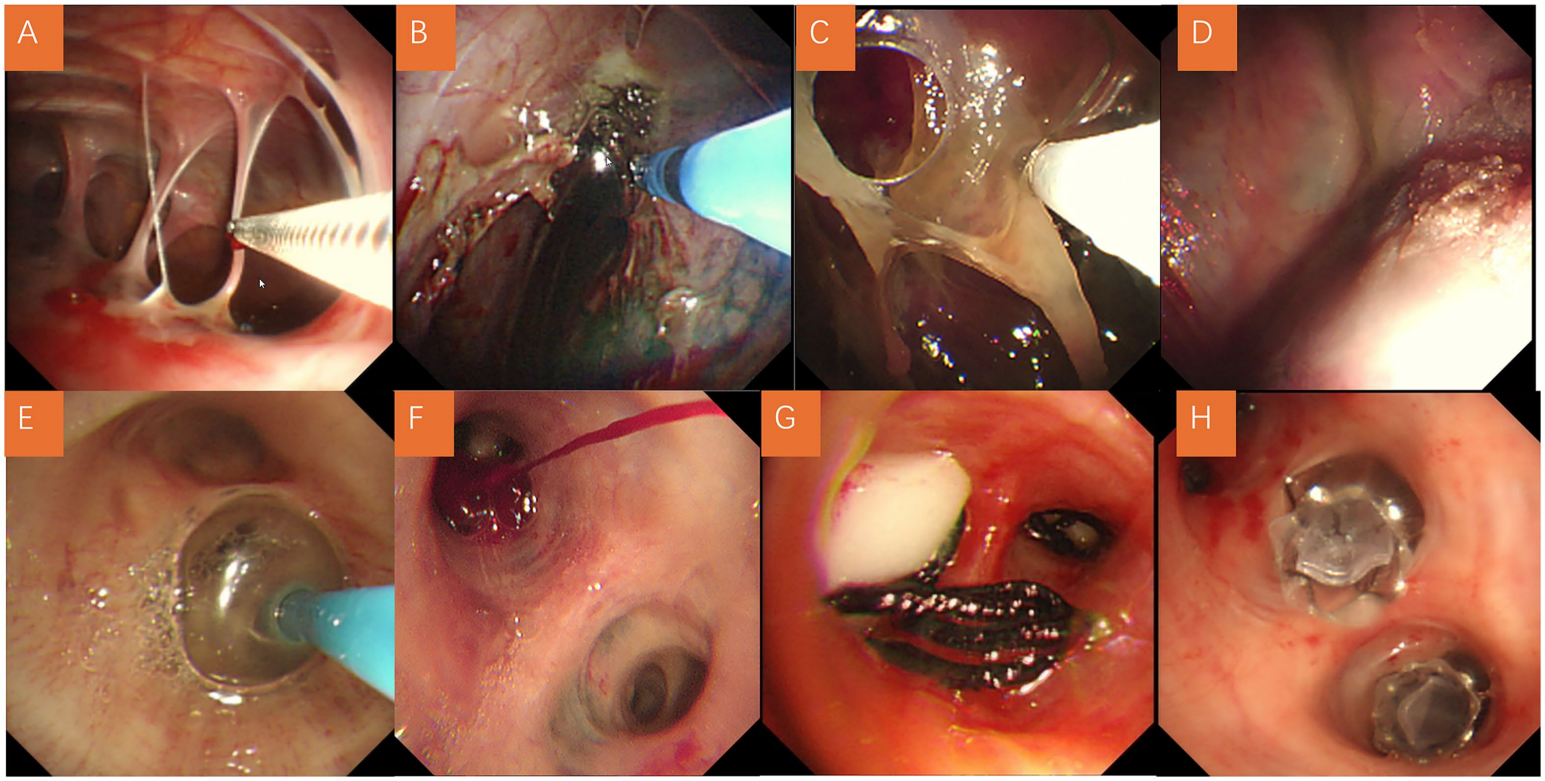
Representative interventional procedures in patients with pneumoconiosis-associated pneumothorax. **(A)** Adhesiolysis performed under medical thoracoscopy. **(B)** Argon plasma coagulation (APC) applied under thoracoscopy. **(C)** Cryotherapy under medical thoracoscopy. **(D)** Instillation of medical adhesive under thoracoscopy. **(E)** Bronchoscopic balloon occlusion for localization of air leak. **(F)** Bronchoscopic autologous blood instillation for selective bronchial occlusion. **(G)** Placement of a silicone plug for bronchial occlusion. **(H)** Placement of an endobronchial one-way valve.

Management strategies were individualized based on pneumothorax severity, recurrence, and comorbid conditions. All patients were administered oxygen therapy during their hospital stay. The majority of patients (37/42, 88.1%) underwent chest tube drainage, which served as the primary treatment. The overall median duration of chest tube placement was 6.5 days (IQR, 2–12). Most pneumothoraces resolved with chest tube drainage, but persistent air leaks (PAL >7 days) required additional interventions such as pleurodesis, medical thoracoscopy, or bronchoscopic occlusion. Overall, 50% of patients (21/42) achieved resolution within 7 days and 76.1% (32/42) within 14 days, whereas those with persistent air leaks lasting more than 7 days were considered clinically significant and required further intervention (15). Three patients underwent medical thoracoscopy with bullectomy and/or pleurodesis, particularly when bullae were clearly identified on imaging or when conservative methods failed (16). We applied a combined pleurodesis technique by inserting a supplementary small-bore catheter (size 8F) above the primary chest tube. Through this route, hypertonic glucose and thrombin were instilled twice weekly (17), which facilitated pleural adhesion and resolution of persistent air leaks. Outcomes following medical thoracoscopy were generally favorable, with no immediate recurrence (Figure 2). In selected cases with persistent air leaks (7/42, 16.7%), selective bronchial occlusion was attempted using balloon occlusion, autologous blood instillation, silicone plugs, or endobronchial one-way valves (14, 18) (Figure 3). These procedures were mainly performed in patients with persistent air leaks after chest tube drainage or poor surgical candidacy due to severely impaired pulmonary function, and were generally associated with short-term improvement in air leak control during hospitalization. Although performed in a minority, these approaches highlighted the value of interventional pulmonology in managing high-risk patients who were poor surgical candidates, mainly due to severely impaired pulmonary function. Given the high prevalence of infection, 32 patients (76.2%) received antibiotic treatment, most commonly cephalosporins, fluoroquinolones, or carbapenems (Table 3).

**Table 3:** Management strategies and outcomes in patients with pneumoconiosis-related pneumothorax (*N* = 42).

Management modality	Numbers (*n*/%)
Chest tube drainage	37 (88.1%)
Medical thoracoscopy^#^	3 (7.1%)
Bronchoscopic interventions^#^	7 (16.7%)
Antibiotic therapy	32 (76.2%)
Other conservative management^$^	5 (11.9%)
Time to resolution	
≤ 7 days	21 (50%)
7 days < Time to resolution ≤15 days	11 (26.2%)
>15 days	10 (23.8%)
Overall outcome	
Improved/discharged	37 (88.1%)
Transferred*	2 (4.8%)
Discharged against advice	2 (4.8%)
Discharged with chest tube	1 (2.4%)

#Procedures were mainly performed in patients with persistent air leaks after chest tube drainage or poor surgical candidacy due to impaired pulmonary function $: Other conservative management refers to oxygen therapy, observation, and supportive treatment without additional invasive interventions such as pleurodesis, thoracoscopy, or bronchoscopic occlusion. *As the patients were diagnosed with tuberculosis, they were referred to a tuberculosis hospital.

The average hospital stay was 14.8 ± 6.7 days (range, 3–22 days). Of the 42 patients, 37 (88.1%) showed clinical improvement and were discharged home after successful resolution of pneumothorax. Two patients were transferred to a tuberculosis-specialized facility due to concomitant active tuberculosis requiring focused management. Follow-up revealed that their chest tubes were successfully removed after 18 and 25 days of drainage, respectively. Two patients were discharged against medical advice, primarily due to financial constraints and perceived poor prognosis, and both were subsequently lost to follow-up. One patient was discharged with a chest tube in place for ongoing outpatient management of a persistent air leak after 12 days of hospitalization and 12 days of chest tube placement. Follow-up 1 month later revealed that the chest tube had been removed at a local hospital after a total of 35 days in place (Table 3). No in-hospital deaths were recorded in this cohort. Notably, patients with stage III pneumoconiosis and concurrent infection were more likely to experience prolonged hospitalization and complications such as persistent air leak.

Recurrent pneumothorax patients had significantly longer chest tube duration (median 15.5 vs. 4.5 days; U = 47.0, *p* = 0.0043) and longer hospital stay (median 19.5 vs. 9.0 days; U = 41.0, *p* = 0.0024) compared with first-time cases. The median interval between the recurrent and previous pneumothorax was 47.5 days (IQR, 26.3–82.5 days). Pleurodesis (37.5% vs. 20.6%), medical thoracoscopy (12.5% vs. 5.9%), and bronchoscopic occlusion (37.5% vs. 11.8%) were all more frequent in recurrent cases, but differences were not statistically significant (all Fisher *p* > 0.05). Antibiotic therapy was commonly used in both groups (87.5% vs. 73.5%), with no significant difference (Fisher *p* = 0.326; Table 4).

**Table 4:** Comparison of clinical features and management: first-time vs. recurrent pneumothorax.

Variable	First-time pneumothorax (*n* = 34)	Recurrent pneumothorax (*n* = 8)	U/*χ*^2^ value	*p* value
Chest tube duration, median (IQR), days	4.5 (0.5–9.0)	15.5 (11.75–22.5)	47.0	0.0043
Hospital stay, median (IQR), days	9.0 (6.0–15.0)	19.5 (11.5–24.5)	41.0	0.0024
Pleurodesis, *n* (%)	7 (20.6%)	3 (37.5%)	NA	Fisher *p* = 0.369
Medical thoracoscopy, *n* (%)	2 (5.9%)	1 (12.5%)	NA	Fisher *p* = 0.432
Bronchoscopic occlusion, *n* (%)	4 (11.8%)	3 (37.5%)	NA	Fisher *p* = 0.094
Antibiotic therapy, *n* (%)	25 (73.5%)	7 (87.5%)	NA	Fisher p = 0.326
The time since the last pneumothorax (IQR), days	NA	47.5 (26.3–82.5)	NA	NA

IQR, Interquartile Range.

## Discussion

Our results are consistent with prior reports from high-burden regions in China, which identified pneumothorax as a frequent complication in progressive pneumoconiosis (5). Previous studies have reported pneumothorax as a complication of silicosis, particularly in patients with high-intensity silica exposure such as denim sandblasting (19), as well as rare cases of bilateral spontaneous pneumothorax in chronic silicosis (20). In contrast, most patients in our cohort had mixed dust pneumoconiosis (73.8%), which may differ from pure silicosis in exposure composition and fibrosis patterns and could influence susceptibility to pneumothorax. Our study extends these observations by providing detailed real-world data from a tertiary referral hospital, where more complex cases are concentrated. The predominance of stage III disease in our cohort (92.9%) highlights the referral bias inherent to tertiary centers (21), and underscores that the burden of pneumoconiosis-related pneumothorax is greatest in patients with severe fibrosis (22) and poor pulmonary reserve (23).

The CT findings in pneumoconiosis-related pneumothorax differ from those typically seen in SSP of other etiologies. Our patients frequently exhibited dense pleural adhesions and irregularly shaped pneumothorax cavities. In some cases, encapsulated pneumothorax developed, which complicated management and necessitated placement of multiple chest tubes in different regions for effective drainage. These imaging and clinical features reflect the extensive pleural fibrosis and architectural distortion characteristic of advanced pneumoconiosis and highlight the technical challenges in managing pneumothorax in this population.

One of the most notable findings of this study was the high prevalence of pulmonary infection, documented in 76.2% of patients. Infections were associated with elevated white blood cell counts and radiographic features such as consolidation and cavitation. The frequent occurrence of infection is likely multifactorial: impaired clearance due to fibrosis and immune dysregulation associated with chronic lung disease (24, 25). These observations highlight the need for rigorous infection control and targeted antimicrobial therapy in this high-risk population. The high prevalence of pulmonary infection in this cohort also suggests that infection may contribute to delayed lung re-expansion, persistent air leaks, and prolonged hospitalization, thereby increasing the overall complexity of pneumothorax management in patients with advanced pneumoconiosis. In addition, patients with concurrent infection appeared more likely to require prolonged chest tube drainage and extended hospitalization, suggesting that infection may represent a marker of disease severity and management complexity in pneumoconiosis-associated pneumothorax. However, because infection status was not evaluated as an independent predictor in adjusted analyses, infection may represent a marker of disease severity and management complexity in pneumoconiosis-associated pneumothorax.

Management in our cohort was heterogeneous and individualized. Chest tube drainage remained the mainstay of treatment (88.1%), consistent with international practice guidelines (12). However, chest tube drainage alone was frequently insufficient, particularly in patients with recurrent pneumothorax or persistent air leaks. According to published reports, 61% of air leaks in SSP resolve within 7 days and 79% within 15 days (26), which is considerably higher than in our cohort of pneumoconiosis-related pneumothorax, where 50% resolved within 7 days and 76.1% within 14 days. This discrepancy likely reflects the greater degree of pleural fibrosis, adhesions, and distorted lung architecture in pneumoconiosis, making spontaneous resolution less likely and necessitating more frequent use of pleurodesis, thoracoscopy, or bronchoscopic interventions. Pleurodesis was performed in a subset, especially in patients unsuitable for surgery. Medical thoracoscopy was applied in three patients (7.1%) with favorable outcomes, though surgical candidacy was often limited by poor pulmonary function. Notably, bronchoscopic occlusion techniques were attempted in 16.7% of patients, primarily for persistent leaks in poor surgical candidates. These minimally invasive approaches are underreported in pneumoconiosis but represent a promising adjunct in the multidisciplinary management of this condition.

Subgroup analysis revealed clinically meaningful differences between first-time and recurrent pneumothorax. Patients with recurrent events had significantly longer chest tube durations (median 15.5 vs. 4.5 days; U = 47.0, *p* = 0.0043) and hospital stays (median 19.5 vs. 9.0 days; U = 41.0, *p* = 0.0024) than those with first-time events. Although pleurodesis, medical thoracoscopy, and bronchoscopic occlusion were more frequently used in recurrent cases, the differences were not statistically significant, likely due to the small sample size. These treatment trends, however, reflect the greater complexity of recurrent pneumothorax, where additional interventions are often required. The high and comparable rates of antibiotic use in both groups underscore the pervasive role of infection in pneumoconiosis-related pneumothorax.

These findings emphasize that recurrent pneumothorax represents a more challenging clinical scenario, often requiring escalated interventions and prolonged hospitalization (27). The longer chest tube duration and hospital stay observed in this subgroup are therefore more likely to reflect greater underlying structural lung damage, pleural adhesions, and persistent air leaks rather than an independent causal effect of recurrence itself.

The strengths of this study include systematic evaluation of a relatively large single-center cohort, detailed subgroup analysis, and incorporation of advanced interventional techniques such as bronchoscopy and medical thoracoscopy. These features enhance the real-world relevance of our findings. However, several limitations must be acknowledged. The retrospective design limits causal inference, and the single-center setting may reduce generalizability, as patients admitted to referral centers typically represent the most severe cases. In addition, because FiO₂ levels were not consistently documented at the time of arterial blood gas sampling, the interpretation of PaO₂ values and the reported prevalence of hypoxemia should be interpreted with caution. Furthermore, the relatively small sample size of the recurrent pneumothorax subgroup limited the statistical power of subgroup comparisons and may have reduced the ability to detect significant between-group differences. Because of the relatively small sample size, particularly in subgroup analyses, multivariable regression was not performed, and potential confounding factors such as smoking status, disease stage, and infection could not be fully adjusted for. Therefore, the findings of this study should be interpreted as exploratory and hypothesis-generating rather than confirmatory, and require validation in larger prospective multicenter studies with adjusted analyses. These methodological constraints limit the generalizability and clinical applicability of the present findings and should be considered when interpreting the results.

Future multicenter prospective studies with adjusted analyses and longer follow-up are needed to validate these findings and better define prognostic factors in pneumoconiosis-associated pneumothorax. Comparative studies are warranted to evaluate pleurodesis agents, surgical and bronchoscopic techniques, while randomized trials of endobronchial valves could provide high-quality evidence. Integration of pulmonary rehabilitation and structured follow-up may further improve outcomes. More broadly, reducing dust exposure and ensuring early intervention remain critical public health priorities.

## Conclusion

Pneumothorax in pneumoconiosis occurs mainly in advanced-stage disease and is frequently complicated by infection. Chest tube drainage remains the cornerstone of therapy, while medical thoracoscopy and bronchoscopic occlusion are valuable in patients with persistent air leaks (PAL >7 days). Recurrent pneumothorax was associated with prolonged hospitalization and longer chest tube duration, underscoring the need for early detection, individualized treatment, and rigorous infection control.

## Data Availability

The raw data supporting the conclusions of this article will be made available by the authors, without undue reservation.
