# Supplementary material for: Clinical characteristics and in-hospital outcomes of pneumothorax in pneumoconiosis: a tertiary referral cohort study
Source: Front Med (Lausanne). 2026 Apr 29;13:1834578. doi: 10.3389/fmed.2026.1834578 (PMC13168199; doi:10.3389/fmed.2026.1834578)
Supplement: Supplementary file 1 [file Data_Sheet_1.docx]

Supplementary materials

**Clinical characteristics and in-hospital outcomes of pneumothorax in pneumoconiosis: a tertiary referral cohort study**

Ping-Yang Hong, Jing-Huang Cai, Yu-Bing Yan, Jing-Rui Lu, Nai-Shan Zheng, Ling Cai, Yi-Li Lin, Mao-Hong Huang, Xiao-Bin Zhang

Contents

[Figure S1. Representative chest CT images before and after treatment in two patients with pneumoconiosis-associated pneumothorax. 2](#_Toc227148297)

# Figure S1. Representative chest CT images before and after treatment in two patients with pneumoconiosis-associated pneumothorax.


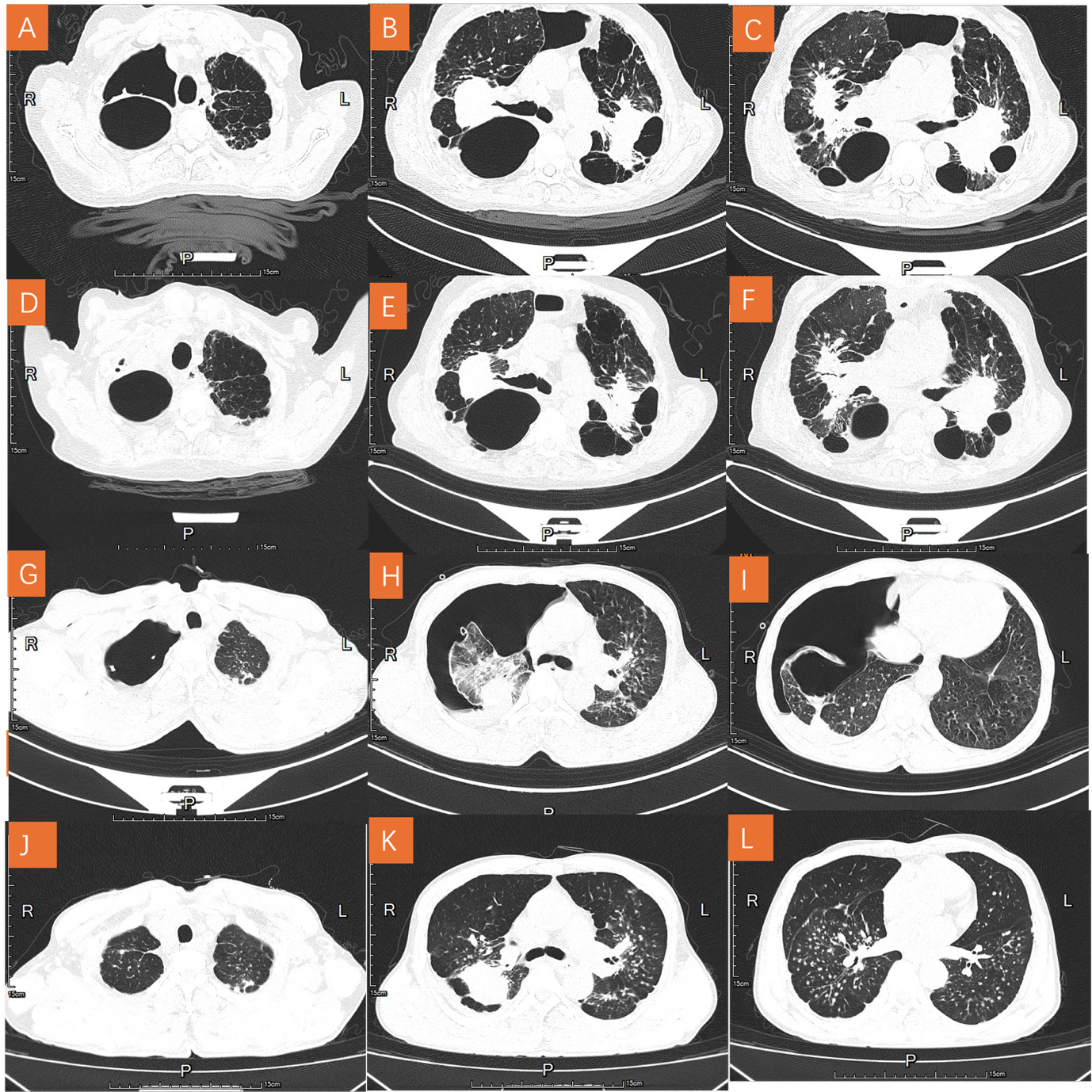


(A-C) Chest CT images of patient A before treatment. (D-F) Chest CT images of patient A after treatment. (G-I) Chest CT images of patient B before treatment. (J-L) Chest CT images of patient B after treatment, showing improvement of pneumothorax and lung re-expansion.
